# Comparison of Anterior Segment Measurements Obtained by Aladdin Optical Biometer and Sirius Corneal Topography

**DOI:** 10.4274/tjo.60476

**Published:** 2016-12-01

**Authors:** Onur Polat, Zeki Baysal, Serkan Özcan, Sibel İnan, Ümit Übeyt İnan

**Affiliations:** 1 Afyonkarahisar State Hospital, Ophthalmology Clinic, Afyonkarahisar, Turkey; 2 Afyon Kocatepe University Faculty of Medicine, Department of Ophthalmology, Afyonkarahisar, Turkey

**Keywords:** Aladdin, optical biometer, anterior segment parameters, Sirius

## Abstract

**Objectives:**

To assess the agreement of anterior segment parameter measurements derived from Aladdin optical biometer using optical low coherence interferometer and Sirius corneal topography using combined Scheimpflug-Placido disk.

**Materials and Methods:**

Data obtained using the Aladdin and Sirius systems from 110 eyes of 59 subjects who had no health problems other than refractive errors were retrospectively evaluated. Anterior chamber depth (ACD), flat (K1) and steep (K2) keratometry readings, and white-to-white distance (WTW) measurements taken with both devices were noted.

**Results:**

The mean age of the patients was 47.31±18.57 years (range, 25 to 79 years). Mean ACD was 3.35±0.4 mm using Aladdin and 3.42±0.44 mm using Sirius. Mean difference in ACD was 0.075 mm greater with Sirius than Aladdin (p<0.001). K1 measurement obtained by Aladdin was an average of 0.409 D higher (p<0.001). No statistically significant differences were detected between the two devices in respect to K2 and WTW measurements (p=0.18, p=0.85 respectively). Pearson correlation analysis showed high correlation between the two devices for all measurements (r=0.985, 0.895, 0.961 and 0.766 for ACD, K1, K2 and WTW respectively; p<0.001).

**Conclusion:**

Anterior segment parameters obtained by Aladdin optical biometer and Sirius anterior segment analysis system correlated well with each other and measurement differences between the devices were clinically negligible except for K1 values.

## INTRODUCTION

The accurate and precise evaluation of anterior segment parameters is critical in order to diagnose many anterior segment diseases, to plan anterior segment surgeries, and to ensure satisfactory postoperative results, patient satisfaction and proper patient management. In recent years, various instruments/techniques including optical coherence tomography, ultrasonic biomicroscopy, Scheimpflug imaging, slit-scanning topography and interferometry have been commonly used in clinical practice to evaluate the anterior segment.^1^

The Aladdin optical biometry instrument (Topcon, Tokyo, Japan) is a new noncontact optical biometry instrument introduced into clinical use in 2012. The device operates on the optical low-coherence interferometry principle and measures axial length (AL), anterior chamber depth (ACD), keratometry, corneal topography, white-to-white distance (WTW) and pupillometry values.^2^

The Sirius topography device (Costruzione Strumenti Oftalmici, Florence, Italy) is an anterior segment analysis system combining Scheimpflug camera and Placido disc technology. This system provides data for corneal thickness, ACD, aqueous depth, lens thickness, keratometry, WTW, pupillography, anterior and posterior corneal topography and corneal wavefront analysis.^3^

There are studies in the literature demonstrating measurement reproducibility for both of these instruments.^2,3,4,5^ However, we were unable to find any published studies examining the agreement between measurements obtained using the two devices. In this study we aimed to compare and assess the agreement between anterior segment parameters measured using the Aladdin optical biometer and data obtained using the Sirius corneal topography system.

## MATERIALS AND METHODS

One hundred ten eyes of 59 healthy subjects who had no pathology other than refractive errors and underwent measurements using both the Aladdin and Sirius devices in our clinic between May 2014 and October 2014 were included in the study and retrospectively evaluated.

Subjects who had a history of ocular surgery, refractive errors greater than ±3 diopters (D), ocular surface problems, topical medication use, or difficulty fixating were not included in the study. The study was designed in accordance with the principles of the Declaration of Helsinki and approval was granted by our departmental ethics committee.

Patients’ demographic data and values for ACD, flat (K1) and steep (K2) keratometry, and WTW obtained using both instruments were recorded.

### Combined Scheimpflug-Placido Disc System (Sirius)

The Sirius topography instrument is an anterior segment analysis system combining a monochromatic 360-degree rotating Scheimpflug camera with a 22-ring Placido disc. Twenty-five radial sections are acquired from the cornea and anterior chamber. The system provides data regarding the tangential and axial curvature of the anterior and posterior corneal surfaces, the global refractive power of the cornea, corneal pachymetry mapping and wavefront analysis. The anterior and posterior surfaces of the cornea are examined using 475 nm blue LED light. While the anterior corneal surface measurements are provided by appropriately combining the Placido and Scheimpflug images, measurements of other interior structures are provided by Scheimpflug imaging.

### Optical Low-Coherence Reflectometry (Aladdin)

The Aladdin optical biometer (Topcon, Tokyo, Japan), introduced into clinical use in 2012, is able to automatically measure biometric parameters such as AL, ACD, keratometry/corneal topography, WTW and pupillometry. AL is measured using an 820 nm superluminescent diode laser. ACD is measured using LED light projected horizontally. The 24-ring Placido disk is used to obtain keratometry and corneal topography measurements. Pupillometry measurements are taken under infrared LED and white LED light to determine photopic and mesopic pupil diameter.

Data were recorded and analyzed using SPSS for Windows 18.0 (SPSS Inc., Chicago, IL, USA). Paired t-test was used to compare data obtained using the two devices. Correlation between the measurements was assessed using Pearson correlation analysis. Evaluations were done between 95% confidence interval and p values less than 0.05 were accepted as statistical significance.

## RESULTS

Of the 59 patients in the study, 33 (55.9%) were women and 26 (44.1%) were men. Mean age was 47.31±18.57 (range, 25-79) years. Mean ACD values were 3.35±0.4 mm as measured by the Aladdin device and 3.42±0.44 mm using the Sirius device; the Aladdin device yielded significantly lower mean ACD values (p<0.001). Mean K1 values were 43.11±1.57 D using the Aladdin and 42.62±1.71 D using the Sirius. K1 measured significantly flatter with the Sirius device (p<0.001). K2 and WTW values measured by Aladdin were 44.04±1.61 D and 11.75±0.47 mm, respectively. In addition, K2 and WTW values measured by Sirius were 44.10±1.65 D and 11.76±0.55 mm, respectively. There were no significant differences in K2 or WTW measurements between the two devices (p=0.183 and p=0.852, respectively).

**Figure 1:**
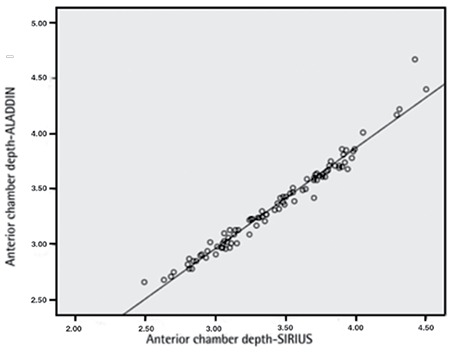
Correlation plot for anterior chamber depth measurements from the Aladdin and Sirius instruments

**Figure 2:**
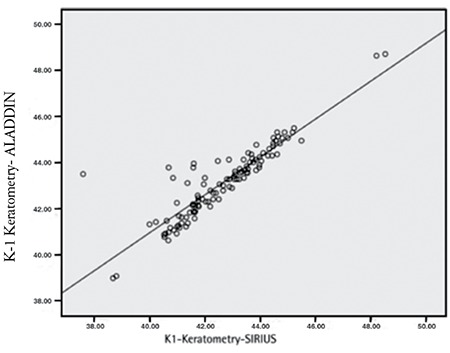
Correlation plot for flat keratometry measurements from the Aladdin and Sirius instruments

The mean differences in Aladdin and Sirius measurements were -0.075±0.08 mm for ACD; 0.409±0.53 D for K1; -0.091±0.37 D for K2; and -0.015±0.33 mm for WTW. There was a high level of correlation between all anterior segment parameter measurements obtained with the two devices (Table 1, Figures 1, 2).

## DISCUSSION

In cataract surgery, currently the most commonly performed procedure, determination of anterior segment parameters is important for the accurate calculation of intraocular lens (IOL) power. Errors in AL, keratometry and ACD measurement have been reported as the most common causes of inaccurate IOL power calculation.^6^ An error of 1 mm in ACD causes postoperative refractive errors of about 1 D in myopic eyes, 1.5 D in emmetropic eyes and 2.5 D in hypermetropic eyes. An error of 0.1 D in keratometry values results in a refractive error of approximately 0.1 D.^7^

In addition to its role in calculating IOL power, ACD is also clinically important for identifying risk of angle closure and detecting anterior segment changes in accommodation and pseudophakic accommodation.^8^ Furthermore, the ACD is one of the factors influencing the accurate determination of optic zone diameter for ablation therapy applied in refractive surgery.^9^ Corneal power, another anterior segment parameter, is important in many critical aspects of refractive surgery planning such as the accurate determination of astigmatism values and axis orientation, power calculation of the IOL to be implanted, and deciding whether corneal astigmatism will be corrected during the same operation.^10,11^ Therefore, it is necessary to assess the accuracy of data from new anterior segment analysis devices by comparing them with those from reference instruments accepted as the gold standard in the measurement of these parameters.

Although conventional A-scan ultrasonography is the gold standard method for measuring ACD, noncontact methods and devices such as partial coherence interferometry, slit-scanning topography, anterior segment optical coherence tomography and Scheimpflug imaging have become widely used in clinics in recent years. Many studies have compared noncontact devices and methods and assessed their reliability and superiority to A-scan ultrasonography in ACD assessment; however, due to the variability in their results, they failed to determine which device or method should be the gold standard in ACD measurement and facilitate standardization.^12,13,14,15,16^ Rabsilber et al.^16^ found a mean ACD of 2.93 mm, while Meinhardt et al.^15^ found a mean ACD of 3.91 mm. Turkish investigators Emre et al.^17^ reported a mean ACD of 3.14 mm in healthy subjects using the Pentacam. Zengin et al.^18^ compared data from ultrasonic biometry and the Orbscan II topography device and reported mean ACD values of 3.05 mm and 3.33 mm, respectively, from the two methods. In another Turkish study, mean ACD was determined to be 3.21 mm using partial optical coherence interferometry and 3.23 mm using optical low-coherence reflectometry.^19^ In the present study, we found mean ACD values of 3.35 mm using the Aladdin device versus 3.42 mm using the Sirius system. These variations in measurements may be a result of differences in the instruments and the methods they use.

In the clinical setting, corneal power measurement used for calculating IOL power is generally performed using an autokeratometer or computerized videokeratography. Many studies have reported that manual keratometry, autokeratometry and corneal topography all yield comparable results in corneal power measurement.^20,21^

Previous studies have also demonstrated that the Aladdin and Sirius devices both provide reproducible measurement.^2,3,4,5^ However, while using the anterior segment parameters measured by these devices it is important to know how their results compare with those of gold standard devices. We found only one study in the literature that utilized the optic biometer (Aladdin) used in the present study.^2^ The authors compared biometric measurements obtained from the Aladdin optical biometry instrument with those of IOL Master, the accepted reference for optic biometric devices, and reported no significant differences between the two devices’ mean ACD and keratometry values.^2^ However, ACD, keratometry values and other anterior segment parameters from the Aladdin optical biometry device must still be compared to those of other devices, especially A-scan ultrasound. Furthermore, studies comparing the reliability of the Sirius system with other Scheimpflug imaging-based devices and instruments using other methods have presented varying results.^6,22,23^

Although we detected a statistically significant difference in the ACD measurements of the Aladdin and Sirius in the present study, this difference is clinically negligible. It is known that when using the Haigis formula, each 0.1 mm change in ACD results in a 0.06 D deviation in the calculated IOL power.^7^ The mean difference in ACD measured by the two devices was -0.075±0.08 (%95 confidence limits: -0.092 and -0.059). Therefore, the 0.07 mm difference between devices is at a clinically acceptable level. In our literature search we found two different studies comparing the Sirius system with Lenstar, another optical biometry instrument. The studies reported differences in ACD values between the devices of -0.10±0.06 mm and -0.07±0.03 mm, thus concluding for the same reason that these differences were negligible in clinical practice.^23,24^ Although it may be negligible, this discrepancy between Aladdin and Sirius measurements may be due to differences in measurement techniques used. Correlation analysis also revealed a high rate of agreement between the measurements obtained using the two instruments.

In the present study, we detected a statistically significant difference of 0.409±0.53 D (95% confidence limits: 0.295 and 0.523 D) in the K1 measurements obtained using the Aladdin and Sirius. An error of 0.1 D in keratometry values causes a refractive error of approximately 0.1 D.^7^ This 0.4 D difference would result in an error of about 0.4 D, which may bring about an undesired and difficult to ignore outcome. In contrast, the difference in K2 values obtained from the two devices was not statistically significant. We were unable to find any information that might explain our finding of significantly different K1 values but comparable K2 values, despite both devices acquiring keratometry measurements from similar paracentral areas (3 mm and 5 mm). Furthermore, correlation analysis showed a high rate of agreement between the K1 and K2 measurements obtained using the two instruments. Although we did not encounter any studies in the literature comparing keratometric analyses of the two devices used in our study, there are various reports using and comparing many different devices and methods in keratometric analysis.^25,26,27^ Some of those studies reported that using certain devices as substitutes for one another may not be suitable due to significant differences in keratometric measurements.^26,27^ As an explanation, the authors suggested that using different methods to measure keratometry may yield different results.

The determination of keratometry values, anterior segment parameters such as ACD and central corneal thickness, as well as WTW is necessary when planning and executing refractive surgery and achieving satisfactory postoperative outcomes. WTW is also utilized in the diagnosis and management of various ocular diseases such as congenital glaucoma, microcornea and megalocornea.^28^ In addition, WTW is important for IOL calculations in modern cataract surgery using third generation formulas to determine haptic dimensions of capsular tension rings and angle-supported IOLs, anterior chamber IOLs and phakic IOLs.^29,30^ We observed no significant difference in the WTW measurements obtained using the Aladdin and Sirius devices and found high correlation between the values.

### Study Limitations

The high correlation between the measurements obtained by these two devices in the present study does not rule out the possibility that those values may be inaccurate. Not using gold standard methods for the measurement of ACD, K1, K2 and WTW in our study and therefore being unable to compare data from the Aladdin and Sirius instruments with those of gold standard devices is a limitation of our study. Our small subject group is another drawback limiting the strength of the study.

## CONCLUSION

Although there were significant differences between the Aladdin and Sirius instruments in the ACD and K1 parameters, there was high correlation between measurements in all studied parameters. The difference in ACD measurements may be clinically negligible, but it may not be appropriate to use these devices interchangeably to measure K1.

### Ethics

Ethics Committee Approval: Retrospective study. Informed Consent: Retrospective study.

Peer-review: Externally peer-reviewed.

## Figures and Tables

**Table 1:**
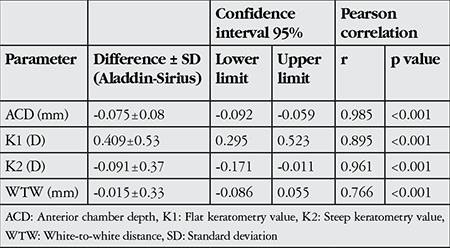
Differences and correlations between anterior segment parameters measured by the Aladdin and Sirius instruments
